# Association of Peripheral Interleukin-6 with Global Cognitive Decline in Non-demented Adults: A Meta-Analysis of Prospective Studies

**DOI:** 10.3389/fnagi.2017.00438

**Published:** 2018-01-08

**Authors:** Steven Bradburn, Jane Sarginson, Christopher A. Murgatroyd

**Affiliations:** ^1^School of Healthcare Science, Manchester Metropolitan University, Manchester, United Kingdom; ^2^NIHR Greater Manchester Primary Care Patient Safety Translational Research Centre, University of Manchester, Manchester, United Kingdom

**Keywords:** inflammation, cognitive aging, inflammaging, interleukin-6, meta-analysis, cognitive decline, longitudinal studies, prospective studies

## Abstract

**Background:** Elevated biomarkers of systemic inflammation have been reported in individuals with cognitive decline, however, most of the literature concerns cross-sectional analyses that have produced mixed results. This study investigates the etiology of this association by performing meta-analyses on prospective studies investigating the relationship between baseline interleukin-6 (IL-6), an established marker of peripheral inflammation, with cognitive decline risk in non-demented adults at follow-up.

**Methods:** We reviewed studies reporting peripheral IL-6 with future cognitive decline, up to February 2017 by searching the PubMed, Science Direct, Scopus and Google Scholar databases. Studies which contained odds ratios (ORs) for the association between circulating baseline IL-6 and longitudinal cognitive performance in non-demented community dwelling older adults were pooled in random-effects models.

**Results:** The literature search retrieved 5,642 potential articles, of which 7 articles containing 8 independent aging cohorts were eligible for review. Collectively, these studies included 15,828 participants at baseline. Those with high circulating IL-6 were 1.42 times more likely to experience global cognitive decline at follow-up, over a 2–7-year period, compared to those with low IL-6 (OR 1.42, 95% CI 1.18–1.70; *p* < 0.001). Subgroup and sensitivity analyses suggests that this association is independent of the study sample size, duration of follow-up and cognitive assessments used.

**Conclusions:** These results add further evidence for the association between high peripheral inflammation, as measured by blood IL-6, and global cognitive decline. Measuring circulating IL-6 may be a useful indication for future cognitive health.

## Introduction

Globally, the number of older adults (≥60 years old) is projected to increase by 56%, from 901 million to 1.4 billion, over the next 14 years and surpass 2 billion by the year 2050 (United Nations, 2015). Analysis involving European studies of aging suggest that the prevalence of cognitive decline in older adults is as high as 28% (Scafato et al., 2010). Some, but not all, of those who experience cognitive decline may go on to develop mild cognitive impairment (MCI). Those with MCI are then at an increased risk of developing dementia (Korolev et al., 2016). The underlying pathogenesis behind this heterogenous transition from cognitive aging to dementia is still under debate but, evidence suggests that systemic inflammation may be a contributing factor (Cunningham and Hennessy, 2015).

Systemic inflammation gradually increases with age, commonly referred to as inflamm-aging. Of the cytokines implemented in the inflamm-aging process, interleukin-6 (IL-6) is regarded as one of the main inflammatory components resulting in the age-associated pathologies (Franceschi and Campisi, 2014). Physiologically, IL-6 is a hormone-like cytokine with pleiotropic capabilities including roles in immunological homeostasis (Hunter and Jones, 2015), such as upregulating acute phase response proteins (e.g., C-reactive protein), and signaling within the central nervous system (CNS) (Spooren et al., 2011; Erta et al., 2012).

Dysregulation of IL-6 has been implicated in the modulation of various cognitive functions (Donzis and Tronson, 2014) and previous meta-analyses have reported associations between upregulated peripheral IL-6 with Alzheimer's disease (AD) (Swardfager et al., 2010; Lai et al., 2017) and postoperative cognitive dysfunction (Peng et al., 2013). These studies report associations between peripheral inflammation during and after the onset of neuropathologies. Exploring these associations longitudinally in the non-demented, however, may provide a better insight into the pathological roles peripheral inflammation plays in the CNS.

The current state of the literature concerning IL-6 and cognitive decline in prospective cohorts of old adults has so far produced mixed findings (Weaver et al., 2002; Yaffe et al., 2003; Dik et al., 2005; Jordanova et al., 2007; Rafnsson et al., 2007; Schram et al., 2007; Singh-Manoux et al., 2014), with some stating a significant association between baseline IL-6 with cognitive decline while others failed to replicate such results. Further, systematic analyses remain inconclusive (Li and Yu, 2017).

The aim of this study was to collate the current data from the literature and perform a meta-analysis for longitudinal studies reporting the association between baseline peripheral IL-6 and future cognitive decline in adults without dementia. This approach is warranted in order to increase the statistical power and thus provide clarity to such an association.

## Methods

### Search strategy

All analyses were performed according to the PRISMA guidelines (Liberati et al., 2009). We searched the published literature in the Scopus, PubMed, Science Direct and Google Scholar databases up to February 2017.

Search terms were as follows: (“Interleukin-6” OR “IL-6”) AND (cognition OR “cognitive decline” OR “cognitive function” OR “cognitive impairment” OR “cognitive loss” OR memory) AND (aging OR aging) AND (health OR healthy) AND (longitudinal OR prospective). We also manually searched any relevant references cited within retrieved articles. A standardized review protocol has not been published.

### Eligibility criteria

Studies were included if they met the following criteria: (i) a prospective cohort design; (ii) cognition performance was used at baseline and at follow-up; (iii) non-demented older subjects at baseline; (iv) IL-6 measured in unstimulated blood (e.g., *ex vivo* blood was not stimulated by lipopolysaccharide); (v) reported odds ratios (ORs) for the association of baseline IL-6 and future global cognitive decline; (vi) the study population consisted of community-dwelling adults; (vii) the article was available in English. Exclusion criteria included: (i) participants with dementia or cognitive impairment were included at baseline; (ii) the association between baseline IL-6 and cognitive decline was not reported; (iii) the study design was cross-sectional or interventional.

Where multiple publications utilized the same cohort source, the study containing the majority of required data was preferred. Where studies reported alternative statistics, as opposed to ORs, authors were contacted via e-mail and the desired logistical analysis was requested. Two authors agreed to this approach (Rafnsson et al., 2007; Schram et al., 2007) and provided ORs. Unsuccessful requests were otherwise discarded from the review.

### Data extraction

All data were reviewed and extracted by two independent investigators (SB and CM). Results were compared and disagreements were settled through discussion.

The following characteristics and data were extracted from each paper: number of subjects at baseline, proportion of females at baseline, age at baseline, length of study follow-up, assessment of global cognition, OR and 95% confidence intervals (CIs) for adjusted model, and confounders adjusted for in the regression analysis. Where studies stratified subjects into more than two groups (e.g., tertiles, quartiles), high and low IL-6 are defined as those in the highest and lowest grouping, respectively. For studies categorizing IL-6 into two groups, via a median split, high and low IL-6 subjects are defined as those above and below the median. For those reporting tertile groupings, those in the second tertile were classed as intermediate IL-6. Where multiple cognitive assessments were used to assess different cognitive domains, we chose the assessment and outcome reflecting global cognitive decline. Where multiple model testing was applied, we extracted the model with the most adjustments for potential confounders.

### Quality assessment

Each paper included within the meta-analyses was subject to quality assessment by two authors via the Newcastle-Ottawa Quality Assessment Scale for cohort studies (Wells et al., 2000). This scale is based on three categories (population selection, comparability and outcome) with a combined maximum score of 9 points. Ratings were compared between authors and disagreements were settled through discussion.

### Statistical analysis

The Review Manager (RevMan; version 5.3.5; Copenhagen, Denmark) software was used to pool the individual studies of interest. Results were deemed statistically significant when *p* < 0.05. Analysis is presented as ORs based on the likelihood of cognitive decline in the highest baseline IL-6 group compared to the lowest group using a random-effects method.

To investigate heterogeneity between studies, we used the I^2^ index which describes the percentage of variation across the studies in the pooled analysis that is due to inconsistency rather than by chance.

*Post-hoc* sensitivity analyses were carried out to investigate the impact of between study methodological difference on the meta-analyses including, follow up time, cohort size, Newcastle-Ottawa Quality Assessment Scale score and IL-6 measurement method and grouping, as appropriate.

Publication bias was assessed through visual inspection of each Begg's funnel plot and the Egger's test. Analysis was performed using the Stata (StataCorp LP; version 14.2; College Station, TX, USA) software.

## Results

### Study selection

**Figure 1:**
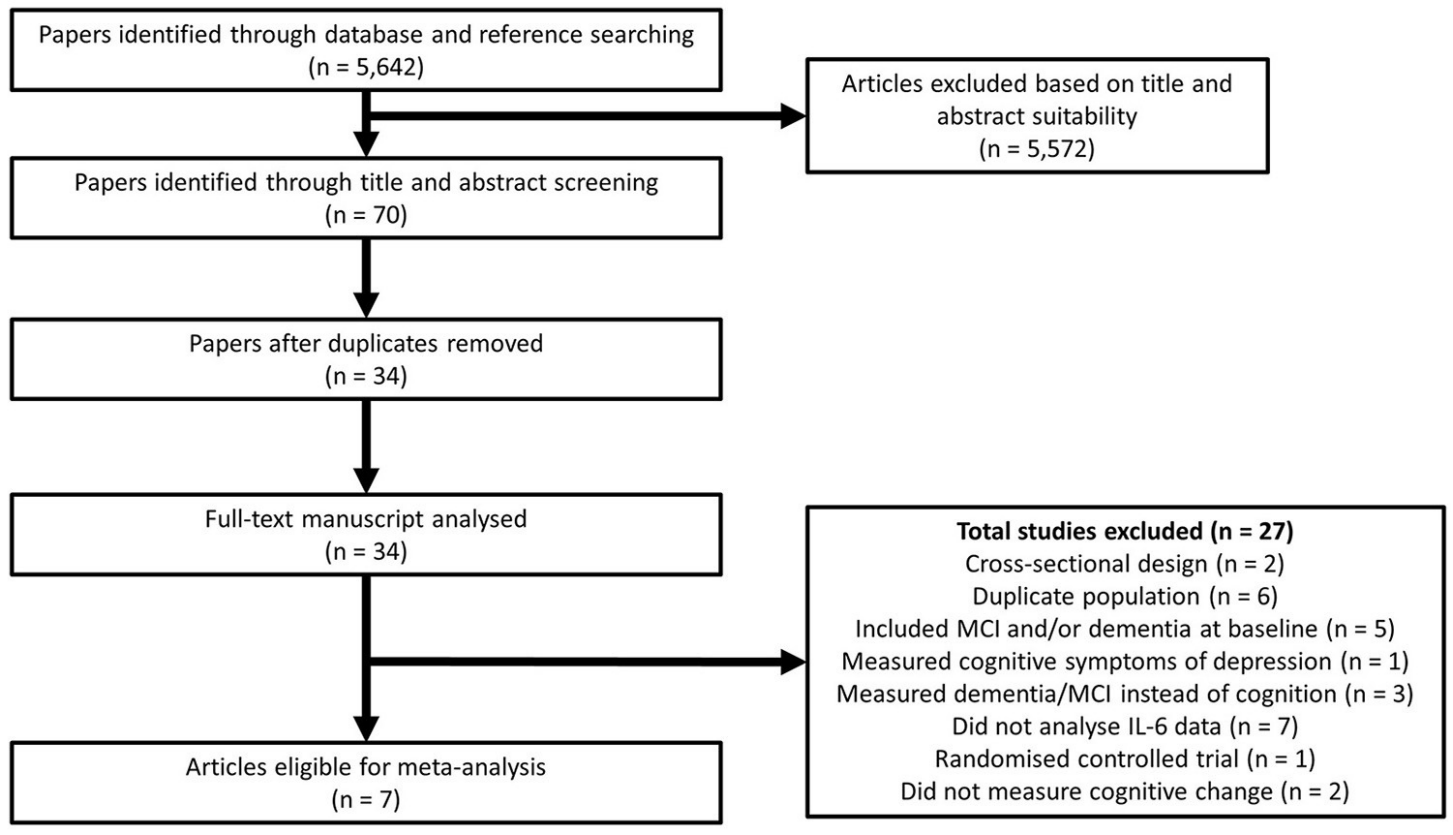
Flowchart summarizing the literature search for the meta-analysis.

Our search strategy returned 5,642 potential articles for inclusion on baseline IL-6 and prospective cognitive functioning, of which 70 were deemed relevant through title and abstract screening (Figure 1). After the removal of duplicate findings, 34 articles remained for full-text analysis. Of these, 27 articles were excluded as they did not meet our inclusion criteria, leaving 7 articles eligible for review. Schram et al. (2007) reported two independent cohorts (Rotterdam and Leiden 85-plus), therefore these were treated as separate studies. Thus, in total 8 studies were included in the review and the study characteristics are described in Table 1. Quality assessment determined that all studies had a high quality score (range: 7–9) (Table 2) with an agreement rate of 86% between author ratings.

**Table 1:** Study characteristics included in the meta-analysis.

**Paper**	**Cohort (location)**	**Subjects at baseline (*n*)**	**Female (%)**	**Mean age at baseline (years)**	**Mean follow up (years)**
Dik et al., 2005	Longitudinal Aging Study Amsterdam (The Netherlands)	1,284	51	75.4 ± 6.6	3
Jordanova et al., 2007	N/A (Britain)	290	57	65.5 ± 5.5	3
Rafnsson et al., 2007	Edinburgh Artery Study (Britain)	452	50	73.1 ± 5.0	4
(Schram et al., 2007) (Leiden 85-Plus cohort)	Leiden 85-Plus cohort (The Netherlands)	491	65	85	3.4
(Schram et al., 2007) (Rotterdam cohort)	Rotterdam cohort (The Netherlands)	3,874	58	72.1 ± 6.9	4.6
Singh-Manoux et al., 2014	The Whitehall II Study (Britain)	5,217	28	55.7 ± 6.0	5
Weaver et al., 2002	The MacArthur Study of Successful Aging (America)	1,189	55	74.3 ± 2.7	7
Yaffe et al., 2003	The Health ABC Study (America)	3,031	52	73.6 ± 2.9	2

**Table 2:** Quality assessment of the included studies via the Newcastle-Ottawa Quality Assessment Scale.

**Study**	**Selection^a^**	**Comparability^b^**	**Outcome^c^**	**Total**
Weaver et al., 2002	^****^	^**^	^**^	8
Yaffe et al., 2003	^****^	^**^	^***^	9
Dik et al., 2005	^***^	^**^	^**^	7
Jordanova et al., 2007	^**^	^**^	^***^	7
Rafnsson et al., 2007	^***^	^**^	^***^	8
(Schram et al., 2007) (Rotterdam cohort)	^****^	^**^	^**^	8
(Schram et al., 2007) (Leiden 85-plus cohort)	^***^	^**^	^***^	8
Singh-Manoux et al., 2014	^**^	^**^	^***^	7

Scores per section are presented as asterisks. ^*^ = one point. Only those answers with an asterisk are given a score.

a Selection; (1) **Representativeness of the exposed cohort (high/intermediate IL-6 group)**: (a) Truly representative of the average community-dwelling older adults^*^. (b) Somewhat representative of the average community-dwelling older adults^*^. (c) Selected group of users e.g., nurses, volunteers. (d) No description of the derivation of the cohort. (2) **Selection of the non-exposed cohort (low IL-6 group)**: (a) Drawn from the same community as the exposed cohort^*^. (b) Drawn from a different source. (c) No description of the derivation of the non-exposed cohort. (3) **Ascertainment of IL-6 category**: (a) Quantified from blood (unstimulated) using an appropriate technique (e.g., ELISA)^*^. (b) No description. (4) **Demonstration that dementia was not present at start of study**: (a) Yes – assessed via interview or using an established cognitive test^*^. (b) No - presumed.

b Comparability; (1) **Comparability of cohorts on the basis of the design or analysis**: a) Study controls for age AND gender in analysis^*^. (b) Study controls for education level or prior cognitive ability in analysis^*^.

c Outcome; (1) **Assessment of outcome**: (a) Cognitive test (e.g., MMSE) which was the same as baseline^*^. (b) Self report. (d) No description. (2) **Was follow-up long enough for outcomes to occur**: (a) Yes (average ≥ 2 years)^*^. (b) No (average < 2 years). (3) **Adequacy of follow up of cohorts**: a) Complete follow up - all subjects accounted for^*^. (b) Subjects lost to follow up unlikely to introduce bias - small number lost (≤25%), or description provided of those lost suggesting no different from those followed^*^. (c) Follow up rate ≤50% and no description of those lost. (d) No statement.

### Study characteristics

In total the studies contained 15,828 participants at baseline. Three studies (Yaffe et al., 2003; Rafnsson et al., 2007; Singh-Manoux et al., 2014) reported proportions of global cognitive decline upon follow-up (8,700 at baseline; 792 declined at follow-up; 9%). All the studies contained a mix of male and female participants. Follow-up periods ranged from 2 to 7 years.

Two studies measured IL-6 from the serum (Dik et al., 2005; Singh-Manoux et al., 2014) and six, including both independent cohorts presented in the Schram et al. study, measured IL-6 from plasma (Weaver et al., 2002; Yaffe et al., 2003; Jordanova et al., 2007; Rafnsson et al., 2007; Schram et al., 2007). All the studies quantified IL-6 from the blood by using an enzyme-linked immunosorbent assay (ELISA) technique.

Assessments of global cognitive functioning consisted of either the Mini-Mental State Examination (MMSE), Modified MMSE (3MS) or a battery of assessments (Table 3). Further, the definition of global cognitive decline differed between studies, however, three of the studies which used the MMSE as the assessment (Schram et al., 2007; Singh-Manoux et al., 2014) had identical definitions (Table 3). All studies included adjustments for age, gender and education at a minimum in their models (Table 3).

**Table 3:** Summary of global cognitive assessments and definitions used in each study included in the meta-analysis.

**Paper**	**Cognitive test**	**Global cognitive decline definition**	**IL-6 categories (pg/mL)**	**Blood specimen**	**Model adjustments**
Dik et al., 2005	MMSE	Change based on cognitive test and regression to the mean.	Low: < 5.0 High: 5.0–58.3	Serum	Age, gender, education.
Jordanova et al., 2007	Battery	Factor analysis based on cognitive score change.	Low: ≤3.1 High: >3.1	Plasma	Age, gender, education, stroke, hypertension, diabetes, smoking, alcohol status, BMI, NSAID use, disability.
Rafnsson et al., 2007	Battery	≥1 standard deviations from a general cognitive factor value.	Low: 0.55–1.66 Intermediate: 1.67–3.00 High: 3.01–100	Plasma	Age, gender, depressed mood, peak prior cognitive ability, lifetime smoking, alcohol intake, presence of major cardiovascular diseases, presence of diabetes mellitus.
(Schram et al., 2007) (Leiden 85-Plus cohort)	MMSE	≥3 points in MMSE scores.	Low: 0–4 Intermediate: 5–25 High: 26–75,001	Plasma	Age, gender, education.
(Schram et al., 2007) (Rotterdam cohort)	MMSE	≥3 points in MMSE scores.	Low: 0.53–1.82 Intermediate: 1.82–3.09 High: 3.10–80	Plasma	Age, gender, education.
Singh-Manoux et al., 2014	MMSE	≥3 points in MMSE scores.	Low: < 1.18 Intermediate: 1.18–1.74 High: ≥1.75	Serum	Age, gender, ethnicity, education, smoking, obesity, Framingham cardiovascular disease risk score, coronary heart disease, stroke, cancer, diabetes antidepressant use.
Weaver et al., 2002	Battery	≥7 points in cognitive score.	Low: < 2.13 Intermediate: 2.13–3.8 High: ≥3.8	Plasma	Age, race, gender, yearly income, education level, alcohol intake, activity level, BMI, self-reported history of cancer or diabetes, HBA1c levels, baseline cognitive scores.
Yaffe et al., 2003	3MS	>5 points in 3MS scores, or if taking cholesterase inhibitor, or hospitalized for dementia.	Low: 0.2–1.4 High: 2.4–16.0	Plasma	Age, education, race, gender, smoking, alcohol use, BMI, self-reported health, co-morbidities, baseline 3MS, use of NSAID.

### Association between high baseline IL-6 and global cognitive decline: meta-analysis

**Figure 2:**
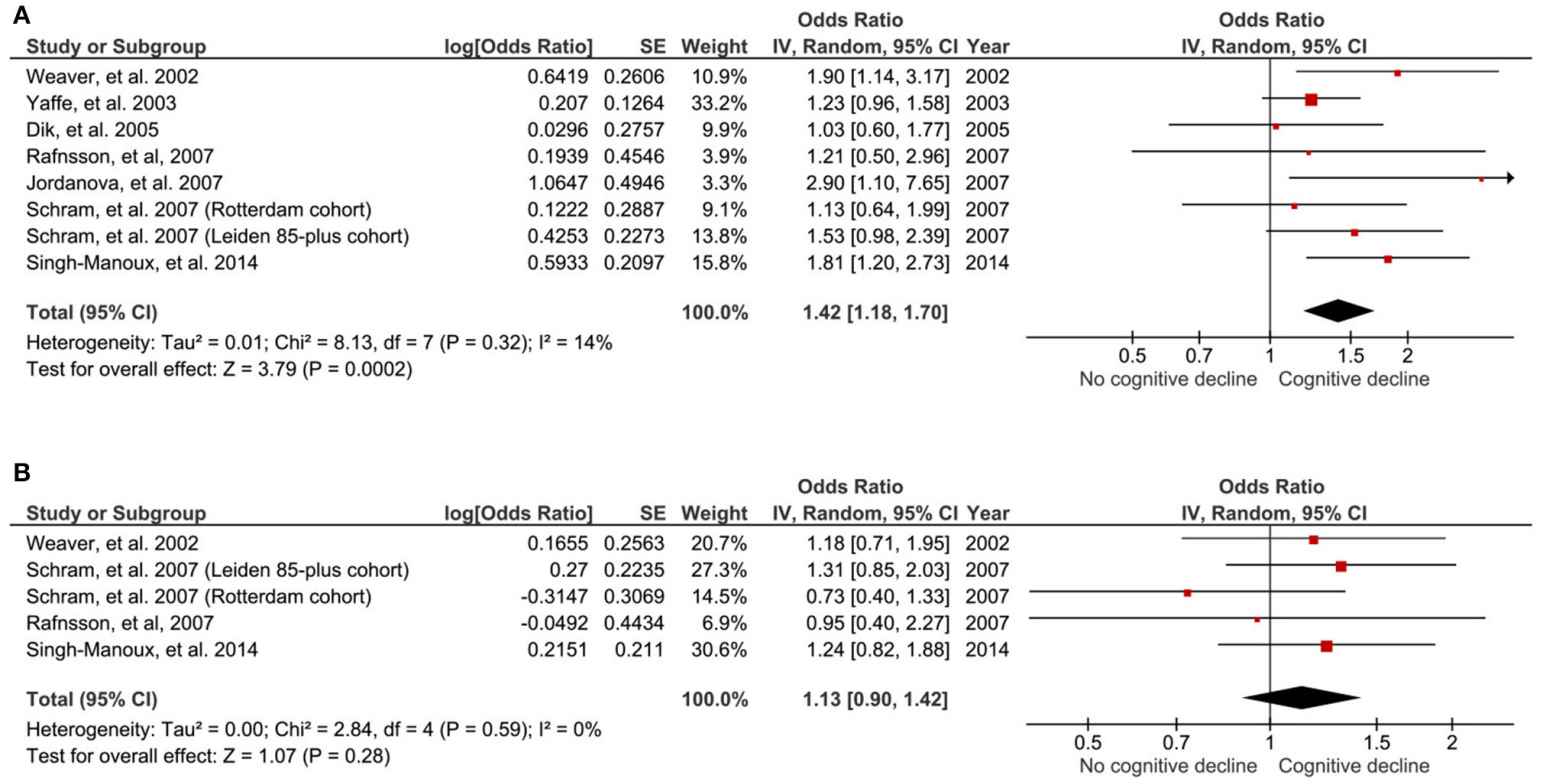
Forest plot for the association between high **(A)** and intermediate **(B)** peripheral levels of interleukin-6 and future global cognitive decline analysis.

Those with high baseline IL-6 were 1.42 times more likely to encounter global cognitive decline at follow-up, compared to those with low IL-6 (OR 1.42, 95% CI 1.18–1.70, *p* < 0.001; Figure 2A). No study heterogeneity was evident (*I*^2^ = 14%, *p* = 0.32; Figure 2A).

### Association between high baseline IL-6 and global cognitive decline: subgroup analysis

Subgroup analysis determined the association was independent of the sample size used (<1,000/≥1,000 participants at baseline), duration of follow-up (<4/≥4 years) and the method used to assess global cognitive performance (MMSE/others) (Table 4). The association, however, was no longer evident when including only studies of lower quality (<8 points). The significance remained after removing studies which categorized IL-6 as above/below median (Weaver et al., 2002; Dik et al., 2005), as opposed to upper and lower grouping (OR 1.41, 95% CI 1.16–1.71; *p* < 0.001; 6 studies) and those which used serum to measure IL-6 (Dik et al., 2005; Singh-Manoux et al., 2014) opposed to plasma (OR 1.39, 95% CI 1.14–1.69; *p* = 0.001; 6 studies).

**Table 4:** Subgroup analyses for the association between high peripheral IL-6 and global cognitive decline analysis.

		**Main effect**					**Heterogeneity**			
	**Studies, *n***	**Odds ratio**	**95% CI**		***Z***	***p***	**χ^2^**	**df**	**p**	***I*^2^**
**DURATION OF FOLLOW-UP**										
<4 years	4	1.33	1.03	1.73	2.15	0.03	4.05	3	0.26	26%
≥4 years	4	1.59	1.22	2.08	3.44	<0.01	2.60	3	0.46	0%
**SAMPLE SIZE AT BASELINE**										
<1,000	3	1.61	1.12	2.33	2.54	0.01	1.85	2	0.40	0%
≥1,000	5	1.37	1.10	1.72	2.79	<0.01	5.56	4	0.23	28%
**METHOD USED TO ASSESS COGNITION**										
MMSE	4	1.41	1.09	1.82	2.62	<0.01	3.43	3	0.33	13%
Others	4	1.51	1.07	2.13	2.37	0.02	4.67	3	0.20	36%
**QUALITY SCORE**										
<8	3	1.61	0.97	2.67	1.85	0.06	4.34	2	0.11	54%
≥8	5	1.34	1.11	1.61	3.10	<0.01	2.99	4	0.56	0%

*CI, Confidence interval; MMSE, Mini Mental State Examination*.

### Association between high baseline IL-6 and global cognitive decline: risk of bias analysis

**Figure 3:**
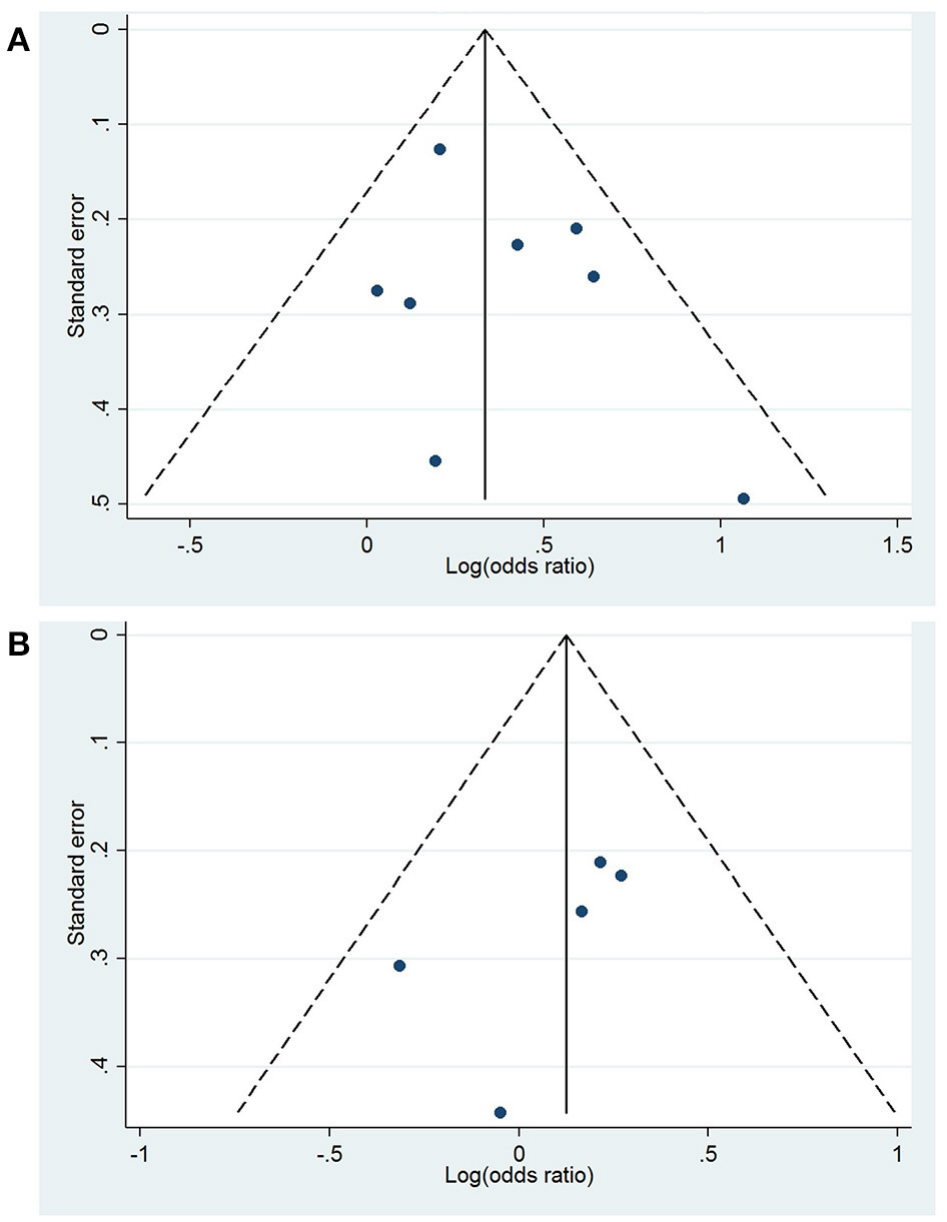
Begg's funnel plots for the association between high **(A)** and intermediate **(B)** peripheral levels of interleukin-6 and future global cognitive decline analysis. Funnel plot displays the log(odds ratio) against the standard error, with the pooled effect (solid line) and pseudo 95% confidence limits (dashed lines).

No publication bias was evident based on visual inspection of Begg's funnel plot (Figure 3A) or through an Egger's test (*p* = 0.379).

### Association between intermediate baseline IL-6 and global cognitive decline: meta-analysis

Five of the aforementioned studies (Weaver et al., 2002; Rafnsson et al., 2007; Schram et al., 2007; Singh-Manoux et al., 2014) also provided data for the association of cognitive decline in those with intermediate (second tertile group) baseline concentrations of IL-6. There was no significant association between those with intermediate baseline IL-6 and the likelihood of cognitive decline at follow-up, compared to those with low IL-6 (OR 1.13, 95% CI 0.90–1.42; *p* = 0.28; Figure 2B). No significant study heterogeneity (I^2^ = 0%, pP = 0.59; Figure 2B) was evident.

### Association between intermediate baseline IL-6 and global cognitive decline: risk of bias analysis

No publication bias was observed when visually inspecting the Begg's funnel plot (Figure 3B) and through an Egger's test (*p* = 0.216).

## Discussion

Individually, many studies included within these analyses did not report significant findings. However, from a collective analysis containing 15,828 older adults who were community-dwelling at baseline, those with high baseline IL-6 were 1.42 times more likely to develop global cognitive decline during a follow-up period of 2–7 years, compared to those with low IL-6.

This is the first meta-analysis to investigate baseline IL-6 and longitudinal global cognitive decline in non-demented adults. Our results suggest that there is an association with high, but not intermediate, baseline concentrations of IL-6 and an increased likelihood of global cognitive decline upon follow-up. Interestingly, three of the included studies (Schram et al., 2007; Singh-Manoux et al., 2014) defined cognitive decline as a change of ≥3 points in MMSE scores between baseline and follow-up. The MMSE is a popular cognitive assessment frequently utilized in large, population based cohorts. It has previously been suggested that a change in MMSE scores of between 2 and 4 points indicated a reliable change at the 90% confidence level in non-demented older adults (Stein et al., 2012). Based on this and to reduce heterogeneity between studies, it may be useful if future studies adopt this definition when the MMSE is applied to define cognitive decline.

Mechanisms underlying bidirectional neuro-immune interactions are becoming increasingly understood (Engelhardt et al., 2017; Pavlov and Tracey, 2017) with numerous routes into the CNS being documented. For example, IL-6 has been shown to directly cross the BBB in a murine model, albeit at a low level, via a saturable transport system (Banks et al., 1994). It is also proposed that peripheral cytokines may have greater access to the CNS through the circumventricular organs (CVOs), brain regions which have a high permeability to the circulating milieu. For example, in rats peripheral injections of IL-6 resulted in the activation of cells within the CVOs (Harré et al., 2003), suggesting a potential pathway from periphery to CNS. Other routes into the CNS include indirect signal propagation at the BBB (Eskilsson et al., 2014). There is also a significant relationship between IL-6 concentrations in the plasma and cerebrospinal fluid (CSF) in those with AD (Sun et al., 2003) and a lagged correlation has been observed in healthy subjects (Agorastos et al., 2014). It is also possible that the aged CNS is more susceptible to peripheral inflammatory cytokines. For example, Montagne and colleagues have shown that the BBB in humans becomes damaged and permeability is increased within regions responsible for learning and memory, such as the hippocampus, during aging (Montagne et al., 2015). Additionally, recurrent insults toward the BBB, due to recurrent infections or even increased exposure to exogenous IL-6 (de Vries et al., 1996), have also been shown to increase BBB permeability (Varatharaj and Galea, 2017). Therefore, it is possible that BBB dysfunctions, both age-associated and the presence of underlying infections, may be present within these populations. Taken together, there is potential for peripheral IL-6 to gain access into the CNS and this is further exacerbated during aging.

The periphery to CNS routes raises the possibility of peripheral IL-6 as a contributing factor toward neuroinflammation. Conversely, raised IL-6 levels in the circulation may merely be a reflection of the neuroinflammatory processes occurring during neurodegeneration and cognitive decline. For example, in rats it has been shown that IL-6 is secreted from the brain following intracerebroventricular injections of labeled IL-6 (Chen et al., 1997) and following a cerebroventricular inflammatory insult (Romero et al., 1996). Further, in humans, the brain has been shown to secrete IL-6 into the circulation following prolonged exercise (Nybo et al., 2002). Considering the plethora of sources of IL-6 production within the CNS, including astrocytes, microglia and neurones and that IL-6 is upregulated during neurodegeneration (Erta et al., 2012), it could be possible that some CNS-derived IL-6 may spill over into the periphery. To elaborate on the cause or effect conundrum, future studies should explore the effect of chronic low-grade increases in peripheral IL-6 levels and the effect this has on the CNS *in vivo*.

Physiologically, IL-6 is essential for developmental functions within the CNS including promoting neural differentiation, modulating adult neurogenesis and controlling neurotrophic expression (Erta et al., 2012). However, prolonged and exacerbated IL-6 exposure to the brain has been associated with numerous neuropathological outcomes. For example, incubating rat hippocampal precursor cells *in vitro* with recombinant IL-6 reduced neurogenesis by approximately 50% and increased the number of apoptotic cells (Monje et al., 2003). Furthermore, the overproduction of IL-6 by astroglia in transgenic mice decreased neurogenesis rates within the dentate gyrus by 63% as well as reducing the proliferation, survival and differentiation of neural progenitor cells (Vallières et al., 2002). Corroborating this, and complementing our findings, research utilizing magnetic resonance imaging has found strong associations between high blood IL-6 concentrations with hippocampal gray matter volumes (Marsland et al., 2008; Satizabal et al., 2012), total brain volumes (Jefferson et al., 2007; Satizabal et al., 2012) and an increased rate of cortical thinning over time (McCarrey et al., 2014). These results suggest that long-term exposure of the brain to increased IL-6 can directly impede neurogenesis and neuronal health, which may manifest into cognitive decline.

The current findings that non-demented older adults with high baseline IL-6 are at an increased risk of future cognitive decline could make it possible of identifying at risk individuals. To bring this into a clinical setting, a considerable amount of methodological standardization is required. Firstly, IL-6 peripheral measures are heavily influenced by a circadian rhythm resulting in considerable inter- and intra-subject variability (Agorastos et al., 2014). Further, as noted in our literature analysis, other parameters such as, the type of commercially available ELISA used, the type of collection tubes used for blood collection, and the fasted state of participants, all varied between studies. These methodological issues have been shown to influence cytokine measurements in clinical research (Zhou et al., 2010) and is the reason why we chose to look at categorically defined IL-6 levels as opposed to continuous measures. Exactly what the specific concentrations of IL-6 classed as “high” and “low” will become clearer once these methodological discrepancies have been addressed.

## Limitations

Despite high quality of the studies used in these analyses, we do accept that there are a few limitations. Firstly, the methodological designs varied considerably between the studies included. We anticipated such discrepancies by applying random-effects analysis throughout. Secondly, we cannot rule out publication bias since the power of statistical tests used here to assess publication bias are significantly reduced when there are fewer than 10 studies in the analysis. Also, we cannot rule out confounding effects by underlying factors that are linked to both elevated peripheral IL-6 levels and cognitive decline, such as cardiovascular disease (CVD) (Compté et al., 2013). We did, however, extract the most adjusted models, many included some adjustments for CVD risk (Table 3), which would have minimized such an effect. Additionally, none of the included studies accounted for dietary factors which are known to influence cognition and IL-6 regulation. Recent insights utilizing the Whitehall II cohort indicate an association between dietary patterns, IL-6 concentrations and cognition in older adults (Ozawa et al., 2017). Also measures of obesity, such as BMI, are also known to correlate with peripheral IL-6 concentrations (Charles et al., 2011). Therefore, it would be interesting if future studies expand on this dietary and obesity association. Finally, almost all included studies reported a collated analysis involving a mix of males and females, therefore potential gender effects could not be explored.

## Conclusion

In conclusion, these results add further evidence for the association between high peripheral inflammation, as measured by blood IL-6, with future cognitive decline. Specifically, those with high IL-6 were 1.42 times more likely to experience global cognitive decline, compared to those with low IL-6. Future studies should focus on exploring the use of circulating IL-6 as a biomarker for future cognitive health and standardizing the processing and measuring of this analyte.

## Author contributions

SB: study design, literature search, statistical analysis, quality assessment, writing of the manuscript. JS: quality assessment, writing of the manuscript. CM: literature search, quality assessment, writing of the manuscript.

### Conflict of interest statement

The authors declare that the research was conducted in the absence of any commercial or financial relationships that could be construed as a potential conflict of interest.

## References

[B1] Agorastos A. Hauger R. L. Barkauskas D. A. Moeller-Bertram T. Clopton P. L. Haji U.. (2014). Circadian rhythmicity, variability and correlation of interleukin-6 levels in plasma and cerebrospinal fluid of healthy men. Psychoneuroendocrinology 44, 71–82. 10.1016/j.psyneuen.2014.02.020 24767621

[B2] Banks W. A. Kastin A. J. Gutierrez E. G. (1994). Penetration of interleukin-6 across the murine blood-brain barrier. Neurosci. Lett. 179, 53–56. 10.1016/0304-3940(94)90933-4 7845624

[B3] Charles B. A. Doumatey A. Huang H. Zhou J. Chen G. Shriner D.. (2011). The Roles of IL-6, IL-10, and IL-1RA in obesity and insulin resistance in African-Americans. J. Clin. Endocrinol. Metab. 96, E2018–E2022. 10.1210/jc.2011-1497 21956416 PMC3232609

[B4] Chen G. Castro W. L. Chow H. H. Reichlin S. (1997). Clearance of 125I-labeled interleukin-6 from brain into blood following intracerebroventricular injection in rats. Endocrinology 138, 4830–4836. 10.1210/endo.138.11.5533 9348212

[B5] Compté N. Boudjeltia K. Z. Vanhaeverbeek M. De B. Pepersack T. Tassignon J.. (2013). Increased basal and alum-induced interleukin-6 levels in geriatric patients are associated with cardiovascular morbidity. PLoS ONE 8:e81911. 10.1371/journal.pone.0081911 24244750 PMC3828251

[B6] Cunningham C. Hennessy E. (2015). Co-morbidity and systemic inflammation as drivers of cognitive decline: new experimental models adopting a broader paradigm in dementia research. Alzheimers. Res. Ther. 7:33. 10.1186/s13195-015-0117-2 25802557 PMC4369837

[B7] de Vries H. E. Blom-Roosemalen M. C. M. Oosten M. van de Boer A. G. van Berkel T. J. C. Breimer D. D.. (1996). The influence of cytokines on the integrity of the blood-brain barrier *in vitro*. J. Neuroimmunol. 64, 37–43. 10.1016/0165-5728(95)00148-4 8598388

[B8] Dik M. G. Jonker C. Hack C. E. Smit J. H. Comijs H. C. Eikelenboom P. (2005). Serum inflammatory proteins and cognitive decline in older persons. Neurology 64, 1371–1377. 10.1212/01.WNL.0000158281.08946.68 15851726

[B9] Donzis E. J. Tronson N. C. (2014). Modulation of learning and memory by cytokines: signaling mechanisms and long term consequences. Neurobiol. Learn. Mem. 115, 68–77. 10.1016/j.nlm.2014.08.008 25151944 PMC4250287

[B10] Engelhardt B. Vajkoczy P. Weller R. O. (2017). The movers and shapers in immune privilege of the CNS. Nat. Immunol. 18, 123–131. 10.1038/ni.3666 28092374

[B11] Erta M. Quintana A. Hidalgo J. (2012). Interleukin-6, a Major Cytokine in the Central Nervous System. Int. J. Biol. Sci. 8, 1254–1266. 10.7150/ijbs.4679 23136554 PMC3491449

[B12] Eskilsson A. Mirrasekhian E. Dufour S. Schwaninger M. Engblom D. Blomqvist A. (2014). Immune-induced fever is mediated by IL-6 receptors on brain endothelial cells coupled to STAT3-dependent induction of brain endothelial prostaglandin synthesis. J. Neurosci. 34, 15957–15961. 10.1523/JNEUROSCI.3520-14.2014 25429137 PMC6608482

[B13] Franceschi C. Campisi J. (2014). Chronic inflammation (inflammaging) and its potential contribution to age-associated diseases. J. Gerontol. A Biol. Sci. Med. Sci. 69(Suppl. 1), S4–S9. 10.1093/gerona/glu057 24833586

[B14] Harré E.-M. Roth J. Gerstberger R. Hübschle T. (2003). Interleukin-6 mediates lipopolysaccharide-induced nuclear STAT3 translocation in astrocytes of rat sensory circumventricular organs. Brain Res. 980, 151–155. 10.1016/S0006-8993(03)02923-8 12865171

[B15] Hunter C. A. Jones S. A. (2015). IL-6 as a keystone cytokine in health and disease. Nat. Immunol. 16, 448–457. 10.1038/ni.3153 25898198

[B16] Jefferson A. L. Massaro J. M. Wolf P. A. Seshadri S. Au R. Vasan R. S.. (2007). Inflammatory biomarkers are associated with total brain volume. Neurology 68, 1032–1038. 10.1212/01.wnl.0000257815.20548.df 17389308 PMC2758770

[B17] Jordanova V. Stewart R. Davies E. Sherwood R. Prince M. (2007). Markers of inflammation and cognitive decline in an African-Caribbean population. Int. J. Geriatr. Psychiatry 22, 966–973. 10.1002/gps.1772 17343293

[B18] Korolev I. O. Symonds L. L. Bozoki A. C. (2016). Predicting progression from mild cognitive impairment to Alzheimer's Dementia using clinical, MRI, and plasma biomarkers via probabilistic pattern classification. PLoS ONE 11:e0138866. 10.1371/journal.pone.0138866 26901338 PMC4762666

[B19] Lai K. S. P. Liu C. S. Rau A. Lanctôt K. L. Köhler C. A. Pakosh M.. (2017). Peripheral inflammatory markers in Alzheimer's disease: a systematic review and meta-analysis of 175 studies. J. Neurol. Neurosurg. Psychiatry 88, 876–882. 10.1136/jnnp-2017-316201 28794151

[B20] Li D. Yu F. (2017). Peripheral inflammatory biomarkers and cognitive decline in older adults with and without Alzheimer's Disease: a systematic review. J. Gerontol. Nurs. 43, 53–60. 10.3928/00989134-20170519-01 28556868

[B21] Liberati A. Altman D. G. Tetzlaff J. Mulrow C. Gøtzsche P. C. Ioannidis J. P. A.. (2009). The PRISMA statement for reporting systematic reviews and meta-analyses of studies that evaluate healthcare interventions: explanation and elaboration. BMJ 339:b2700. 10.1136/bmj.b2700 19622552 PMC2714672

[B22] Marsland A. L. Gianaros P. J. Abramowitch S. M. Manuck S. B. Hariri A. R. (2008). Interleukin-6 covaries inversely with Hippocampal grey matter volume in middle-aged adults. Biol. Psychiatry 64, 484–490. 10.1016/j.biopsych.2008.04.016 18514163 PMC2562462

[B23] McCarrey A. C. Pacheco J. Carlson O. D. Egan J. M. Thambisetty M. An Y.. (2014). Interleukin-6 is linked to longitudinal rates of cortical thinning in aging. Transl. Neurosci. 5, 1–7. 10.2478/s13380-014-0203-0 27066268 PMC4824945

[B24] Monje M. L. Toda H. Palmer T. D. (2003). Inflammatory blockade restores adult hippocampal neurogenesis. Science 302, 1760–1765. 10.1126/science.1088417 14615545

[B25] Montagne A. Barnes S. R. Sweeney M. D. Halliday M. R. Sagare A. P. Zhao Z.. (2015). Blood-brain barrier breakdown in the aging human hippocampus. Neuron 85, 296–302. 10.1016/j.neuron.2014.12.032 25611508 PMC4350773

[B26] Nybo L. Nielsen B. Klarlund Pedersen B. Møller K. Secher N. H. (2002). Interleukin-6 release from the human brain during prolonged exercise. J. Physiol. 542, 991–995. 10.1113/jphysiol.2002.022285 12154196 PMC2290444

[B27] Ozawa M. Shipley M. Kivimaki M. Singh-Manoux A. Brunner E. J. (2017). Dietary pattern, inflammation and cognitive decline: the Whitehall II prospective cohort study. Clin. Nutr. Edinb. Scotl. 36, 506–512. 10.1016/j.clnu.2016.01.013 26874911 PMC5381339

[B28] Pavlov V. A. Tracey K. J. (2017). Neural regulation of immunity: molecular mechanisms and clinical translation. Nat. Neurosci. 20, 156–166. 10.1038/nn.4477 28092663

[B29] Peng L. Xu L. Ouyang W. (2013). Role of Peripheral Inflammatory Markers in Postoperative Cognitive Dysfunction (POCD): a meta-analysis. PLoS ONE 8:e79624. 10.1371/journal.pone.0079624 24236147 PMC3827367

[B30] Rafnsson S. B. Deary I. J. Smith F. B. Whiteman M. C. Rumley A. Lowe G. D. O.. (2007). Cognitive decline and markers of inflammation and hemostasis: the Edinburgh Artery Study. J. Am. Geriatr. Soc. 55, 700–707. 10.1111/j.1532-5415.2007.01158.x 17493189

[B31] Romero L. I. Kakucska I. Lechan R. M. Reichlin S. (1996). Interleukin-6 (IL-6) is secreted from the brain after intracerebroventricular injection of IL-1 beta in rats. Am. J. Physiol. 270, R518–R524. 8780215 10.1152/ajpregu.1996.270.3.R518

[B32] Satizabal C. L. Zhu Y. C. Mazoyer B. Dufouil C. Tzourio C. (2012). Circulating IL-6 and CRP are associated with MRI findings in the elderly: the 3C-Dijon Study. Neurology 78, 720–727. 10.1212/WNL.0b013e318248e50f 22357713

[B33] Scafato E. Gandin C. Galluzzo L. Ghirini S. Cacciatore F. Capurso A.. (2010). Prevalence of Aging-Associated Cognitive Decline in an Italian elderly population: results from cross-sectional phase of Italian PRoject on Epidemiology of Alzheimer's disease (IPREA). Aging Clin. Exp. Res. 22, 440–449. 10.3275/6970 20383053

[B34] Schram M. T. Euser S. M. de Craen A. J. M. Witteman J. C. Frölich M. Hofman A.. (2007). Systemic markers of inflammation and cognitive decline in old age. J. Am. Geriatr. Soc. 55, 708–716. 10.1111/j.1532-5415.2007.01159.x 17493190

[B35] Singh-Manoux A. Dugravot A. Brunner E. Kumari M. Shipley M. Elbaz A.. (2014). Interleukin-6 and C-reactive protein as predictors of cognitive decline in late midlife. Neurology 83, 486–493. 10.1212/WNL.0000000000000665 24991031 PMC4141998

[B36] Spooren A. Kolmus K. Laureys G. Clinckers R. De Keyser J. Haegeman G.. (2011). Interleukin-6, a mental cytokine. Brain Res. Rev. 67, 157–183. 10.1016/j.brainresrev.2011.01.002 21238488

[B37] Stein J. Luppa M. Maier W. Wagner M. Wolfsgruber S. Scherer M.. (2012). Assessing cognitive changes in the elderly: reliable change indices for the Mini-Mental State Examination. Acta Psychiatr. Scand. 126, 208–218. 10.1111/j.1600-0447.2012.01850.x 22375927

[B38] Sun Y. X. Minthon L. Wallmark A. Warkentin S. Blennow K. Janciauskiene S. (2003). Inflammatory markers in matched plasma and cerebrospinal fluid from patients with Alzheimer's disease. Dement. Geriatr. Cogn. Disord. 16, 136–144. 10.1159/000071001 12826739

[B39] Swardfager W. Lanctôt K. Rothenburg L. Wong A. Cappell J. Herrmann N. (2010). A Meta-Analysis of Cytokines in Alzheimer's Disease. Biol. Psychiatry 68, 930–941. 10.1016/j.biopsych.2010.06.012 20692646

[B40] United Nations (2015). Population Division World Population Prospects 2015. Available Online at: http://esa.un.org/unpd/wpp/Download/Probabilistic/Population/ (Accessed January 22, 2016).

[B41] Vallières L. Campbell I. L. Gage F. H. Sawchenko P. E. (2002). Reduced hippocampal neurogenesis in adult transgenic mice with chronic astrocytic production of interleukin-6. J. Neurosci. 22, 486–492. 11784794 10.1523/JNEUROSCI.22-02-00486.2002 PMC6758670

[B42] Varatharaj A. Galea I. (2017). The blood-brain barrier in systemic inflammation. Brain Behav. Immun. 60, 1–12. 10.1016/j.bbi.2016.03.010 26995317

[B43] Weaver J. D. Huang M. H. Albert M. Harris T. Rowe J. W. Seeman T. E. (2002). Interleukin-6 and risk of cognitive decline: MacArthur studies of successful aging. Neurology 59, 371–378. 10.1212/WNL.59.3.371 12177370

[B44] Wells G. Shea B. O'Connell D. Peterson J. Welch V. Losos M. (2000). The Newcastle - Scale for Assessing the Quality of Nonrandomised Studies in Meta-Analyses.pdf. Available Online at: http://www.medicine.mcgill.ca/rtamblyn/Readings/The%20Newcastle%20-%20Scale%20for%20assessing%20the%20quality%20of%20nonrandomised%20studies%20in%20meta-analyses.pdf (Accessed October 21, 2016).

[B45] Yaffe K. Lindquist K. Penninx B. W. Simonsick E. M. Pahor M. Kritchevsky S.. (2003). Inflammatory markers and cognition in well-functioning African-American and white elders. Neurology 61, 76–80. 10.1212/01.WNL.0000073620.42047.D7 12847160

[B46] Zhou X. Fragala M. S. McElhaney J. E. Kuchel G. A. (2010). Conceptual and methodological issues relevant to cytokine and inflammatory marker measurements in clinical research. Curr. Opin. Clin. Nutr. Metab. Care 13, 541–547. 10.1097/MCO.0b013e32833cf3bc 20657280 PMC2955626

